# Antimicrobial Resistance Profiles of Bacteria Isolated from Fresh Vegetables in Free State Province, South Africa

**DOI:** 10.3390/foods14122139

**Published:** 2025-06-19

**Authors:** Dineo Attela Mohapi, Tsepo Ramatla, Oriel Thekisoe, Zenzile Peter Khetsha, Jane Nkhebenyane

**Affiliations:** 1 Centre for Applied Food Safety and Biotechnology, Department of Life Sciences, Central University of Technology, 1 Park Road, Bloemfontein 9300, South Africa; dineoattela@gmail.com (D.A.M.); snkheben@cut.ac.za (J.N.); 2 Unit for Environmental Sciences and Management, North-West University, Potchefstroom 2520, South Africa; oriel.thekisoe@nwu.ac.za; 3 Department of Agriculture, Central University of Technology, Free State, Bloemfontein 9300, South Africa; zkhetsha@cut.ac.za

**Keywords:** cabbage, spinach, bacterial contamination, antibiotic resistance, food safety

## Abstract

The important role of antibiotics cannot be overestimated, as human health relies heavily on them for the treatment of infectious diseases. In this study, the antimicrobial susceptibility profiles of pathogens isolated from spinach (*Spinacia oleracea*) and cabbage (*Brassica oleracea*) collected from Free State Province were investigated. A total of 38 isolates representing 10 species, *Enterobacter cloacae* (5.3%), *Staphylococcus aureus* (13.2%), *Micrococcus luteus* (5.3%), *Staphylococcus sciuri* (5.3%), *Acinetobacter haemolyticus* (5.3%), *Burkholderia cepacia* (15.8%), *Pseudomonas luteola* (15.8%), *Escherichia coli* (18.4%), *Citrobacter freundii* (5.3%), and *Serratia marcescens* (10.5%), were confirmed by the Analytical Profile Index (API). We evaluated antibiotic resistance patterns of 38 unduplicated isolates using the disk diffusion method. As a result, *E. coli* (18.4%), *B. cepacia* (15.8%), *P. luteola* (15.8%), *S. aureus* (13.2%), and *S. marcescens* (10.5%), as well as 5.3% each for *E. cloacae*, *M. luteus*, *S. sciuri*, *A. haemolyticus*, and *C. freundii,* showed resistance to tested antibiotics. The majority (84%) of the isolates showed resistance to tetracycline, and penicillin had a value of 71%. A total of 79% of the antibiotic-resistant isolates demonstrated multidrug resistance (MDR) to several classes such as *β*-lactams, chloramphenicol, tetracycline, aminoglycosides, and macrolides. The results highlight the importance of monitoring the microbiological quality of leafy greens as they contain antibiotic-resistant bacteria that could affect human health when consumed.

## 1. Introduction

Antibiotic resistance is highlighted as a global health crisis that best points out and elucidates the “one health” approach. The “one health” approach is outlined and expressed as a multi-disciplinary platform aimed to provide solutions for human, animal, and environmental health [1]. Thus, it is imperative not only to understand antibiotic utilisation, particularly in agriculture, and its impact but to also have an insight into the emergence of antibiotic resistance, including the complex interaction of elements in humans, animals, and the environment. It is also important to gain insight into the emergence of antibiotic resistance, including the complex interaction of elements in leafy agricultural produce. A larger study regarding the determination of the sources of pathogenic bacteria, including antibiotic resistance, is necessary in order to suggest the sources and relevant steps to mitigate the contamination [2].

Antibiotics in animals are utilised as supplements for growth efficiency, to improve health status, as prophylaxis, or to treat infections or diseases [3]. Different studies showed that 30–90% of the antimicrobials administered to animals are excreted as the parent compound in their faeces or urine [4,5]. The contamination of plants by antibiotic residue can also be performed through different mediums employed for making the soil fertile such as the use of fertiliser/manure, biosolids/sludge, and contaminated irrigation water [6]. Other studies have also reviewed the fate and transport of antibiotic residues and antibiotic resistance genes in agroecosystems following land application of manure waste [7,8]. The emergence of antibiotic resistance is an increasing concern worldwide and in healthcare facilities due to the ongoing explosion of antibiotic-resistant bacterial infections [9]. Antibiotic resistance is of great concern as it is associated with morbidity, mortality, and economics [10,11]. Fresh produce is reported to be the source of exposure to various antimicrobial-resistant bacteria, which is of clinical importance [12]. Additionally, vegetables, particularly leafy green vegetables, are not treated with antibiotics but can be contaminated through various contaminants, such as irrigation water and soil amendments, such as biosolids, including fertiliser utilised on crops. Few studies have reported and highlighted the presence of antibiotic-resistant bacteria (ARB) and antibiotic resistance genes (ARGs) in fresh produce [12,13].

Another essential utilisation of antibiotics other than in livestock is the primary target in controlling bacterial diseases in plants [14]. However, due to the inappropriate practice of misusing antibiotics on vegetables from the primary sector, there has been an increase in the development of microbial resistance [15]. Antibiotic compounds such as tetracycline, oxytetracycline, sulfamethazine, sulfamethoxazole, tylosin, trimethoprim, ofloxacin, ciprofloxacin, and amoxicillin can be absorbed by vegetables such as lettuce (*Lactuca sativa*), cabbage (*Brassica oleracea*), and spinach (*Spinacia oleracea*) from the growth media through their roots [16,17].

It is estimated that by the year 2030, the utilisation of antibiotics will increase by 67%, with almost twice this increase in countries such as Russia, China, Brazil, India, and South Africa [18]. Each year in the United States, at least 2 million people become infected with bacteria that are resistant to antibiotics and at least 23,000 people die each year as a direct result of these infections, and many more people die from other conditions that were complicated by an antibiotic-resistant infections [19]. A study based on 2007 data estimated that 386,000 infections due to multidrug-resistant bacteria occurred in Europe during that year, and 25,000 patients died from those infections [20].

Antimicrobial resistance status, specifically in sub-Saharan Africa, is undefined, and this is because of a lack of real-time data recording, surveillance, and regulation [21]. Phares et al. [22] reported poor practices regarding the utilisation of antibiotics as well as inadequate knowledge regarding their effect on the soil ecosystem amongst farmers in Ghana. A systematic review by Tadesse et al. [23] reported that there is a 42.6% gap in unavailable data on antibiotic resistance, particularly in African countries. In South Africa, a few studies have been conducted on fresh produce in Mpumalanga Province [24], North West Province [25,26], and Gauteng Province [27]. However, there is no information available on the antimicrobial susceptibility profile of pathogens isolated from spinach and cabbage in Free State Province, South Africa.

## 2. Materials and Methods

### 2.1. Study Area and Sample Collection

**Figure 1:**
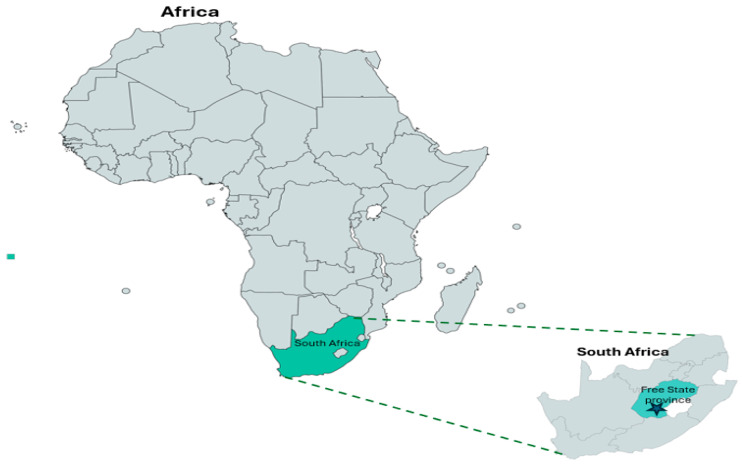
South Africa map showing Free State Province where the samples were collected.

This study was conducted by procuring ninety samples of raw unpackaged spinach [*Spinacia oleracea* (L.)] and ninety samples of cabbage [*Brassica oleracea* var. *capitata* (L.)] heads from five farms, including sixty samples of raw unpackaged spinach phyllosphere and seventy-five samples of cabbage heads from five retail markets, respectively, in different local municipality districts within Free State Province, South Africa (Figure 1). The selected farms represent the major small-scale farms that supply the most leafy greens to various retailers. Spinach and cabbage were chosen due to their minimal processing production, demand, and purchase price. Leafy vegetables, like spinach, are available year round, while cabbage is available during winter, so each province in South Africa is unique in terms of suitable agricultural commodities that can be produced. Additionally, the Free State Agricultural Union reports that the province has 7.515 farming units, the highest in the country. Furthermore, it accounts for 26.4% of South Africa’s field crops and 15.9% of all its livestock. Moreover, Free State Province is responsible for 15% of South Africa’s gross agricultural income. The sector contributes approximately 7% to the provincial gross domestic product. Consumer demand puts pressure on the fresh leafy green vegetable industries for year-round supply. The farms selected are small-scale farms that supply retailers, small villages, street vendors, informal markets, and local supermarkets. Samples were collected in the following towns in Free State Province, South Africa: Motheo District, Mangaung Metropolitan (29.1217 S, 26.2128 E); Lejweleputwa District, Matjhabeng Local Municipality (28.9784 NS, 27.0264 E); Thabo Mofutsanyana District, Setsoto Municipality (28.9093 S, 27.5555 E]); Fezile Dabi District, Moqhaka Local Municipality (27.6373 S, 27.2323 E); and Thabo Mofutsanyana District, Dihlabeng Local Municipality (28.2423 S, 28.3111 E) [28].

### 2.2. Microbiological Techniques and Analysis

A total of 25 g of each collected sample was added to 90 mL of sterile peptone water solution (Merck, SA) and homogenised in a stomacher (Stomacher^®^ 400 circulation Seward, Lasec, SA) for 260 rpm for 1 min. Mashed samples were then filtered through a sterile folded filter paper (Lasec, SA). The sequential dilutions were prepared using filtrated samples for plate count analyses. Subsequently, ten-fold serial dilutions of up to 10^5^ folds of the homogenate were prepared for each sample and utilised for bacterial analysis. Each sample was serially diluted and subsequently analysed in duplicates. Plate count agar (PCA) and selective media, including MacConkey agar with crystal violet and salt, MacConkey agar without crystal violet, Baird–Parker agar supplemented with egg yolk, and violet-red bile (all obtained from Merck, SA) were utilised for culturing [28].

### 2.3. Identification of the Isolates Using API

Colonies were plated on counting agar plates and purified on colony blood agar prior to analysis with API 20E, 20NE, STAPH, and 50 CHB/E for organism identification (bioMérieux, France). Briefly, 1–4 colonies with identical morphology were collected from cultures (18–24 h) and emulsified in 5 mL of sterile sodium chloride (0.85%) for API 20E, 20NE, STAPH, and 50 CHB/E, and the turbidity was adjusted to the equivalent of turbidity of 0.5 McFarland standards. The standardised bacterial suspension was carefully distributed into the test strip tubes to avoid bubble formation. Anaerobiosis was created by overlaying with sterile mineral oil, and the strips were then incubated for 18–24 h at 37 °C in a humid atmosphere. For *Pseudomonadaceae*, an additional oxidase test was performed by adding 2–3 drops of the reagent directly to the suspected colonies on the nutrient agar plate. The colour change was observed within 10 s. When using Kovac’s oxidase reagent, microorganisms are oxidase positive when the colour changes to dark purple within 5 to 10 s [28].

### 2.4. Antibiotic Susceptibility Pattern of the Isolates

The antibiotic susceptibility of the thirty-eight isolates against antimicrobials was determined by the Kirby–Bauer disc diffusion method in Mueller–Hinton Agar (Merck, SA) [29]. The disc diffusion test is a simple, practical, and well-standardised susceptibility method [30,31]. All the isolates were analysed for antimicrobial susceptibility tests against various antibiotic agents. The isolates tested were picked from identified bacteria from farms and retailers. Seven classes of antibiotics were tested, which included β-lactams [penicillin (P; 10 μg), ampicillin (AMP; 10 μg), ceftazidime (CAZ; 30 μg)], aminoglycosides [gentamicin (CN; 10 μg)], chloramphenicol [chloramphenicol (C; 30 μg)], tetracycline [tetracycline (TE; 30 μg)], glycopeptide [vancomycin (VA; 30 μg)], macrolides [erythromycin (E; 15 μg)], and fluoroquinolones [ciprofloxacin (CIP; 5 μg)] (ThermoFisher, South Africa). These antimicrobial agents were selected based on their various pharmacological categories and their availability, which includes their frequency of prescription for the treatment of various bacterial infections in South Africa. The control strains of *E. coli* ATCC 25922 were used to ensure quality control during the antibiotic susceptibility test. Multidrug resistance (MDR) was taken as resistance to three or more classes of antibiotics tested [29]. Following incubation, the zones were measured to the nearest millimeter using a ruler or caliper to include the diameter of the disc in the measurement. A guidelines chart for the interpretation of antibiotic susceptibility was utilised [30].

## 3. Results

### 3.1. Identification of Isolates

A total of thirty-eight non-duplicated isolates (one isolate per sample) were confirmed by API 20E, 20NE, STAPH, and 50 CHB/E, including *E. cloacae* (*n* = 2; 5.3%), *S. aureus* (*n* = 5; 13.2%), *M. luteus* (*n* = 2; 5.3%), *S. sciuri* (*n* = 2; 5.3%), *A. haemolyticus* (*n* = 2; 5.3%), *B. cepacia* (*n* = 6; 15.8%), *P. luteola* (*n* = 6; 15.8%), *E. coli* (*n* = 7; 18.4%), *C. freundii* (*n* = 2; 5.3%), and *S. marcescens* (*n* = 4; 10.5%), and they were recovered from farm and retail spinach and cabbage. Most isolates (60.5%) were of farm origin, with spinach and cabbage contributing 34.2% (*n* = 13) and 26.3% (*n* = 10) of the total, respectively. While fifteen isolates were obtained from the retail markets, nine (23.6%) of these isolates were from spinach and six (15.7%) were from cabbage, as shown in Table 1.

### 3.2. Antibiotic Susceptibility for All Isolates

Analysis of 38 isolates representing 10 species showed that *E. cloacae*, *E. coli*, and *S. marcescens* isolates exhibited resistance to chloramphenicol, while all *E. cloacae*, *S. aureus*, *A. haemolyticus*, *P. luteola*, *E. coli*, *C. freundii,* and *S. marcescens* isolates were resistant to tetracycline. The majority (84%) (*n* = 32) of the isolates showed resistance to tetracycline, followed by penicillin with 71% (*n* = 27). Among the tested antibiotics, vancomycin had the least number of resistant isolates, accounting for only 5.3% (*n* = 2) (Table 2).

Regarding multidrug resistance (MDR), 79% (*n* = 30) of the isolates from fresh vegetables were resistant to three or more classes of antibiotics (Table 3), namely, *β*-lactams (penicillin, ampicillin, ceftazidime), aminoglycosides (gentamicin), chloramphenicol (chloramphenicol), tetracycline (tetracycline), glycopeptide (vancomycin), macrolides (erythromycin), and fluoroquinolones (ciprofloxacin), as well as *E. cloacae* (*n* = 1; 3.2%), *S. aureus* (*n* = 5; 13.2%), *M. luteus* (*n* = 1; 3.2%), *S. sciuri* (*n* = 1; 3.2%), *A. haemolyticus* (*n* = 2; 5.3%), *B. cepacia* (*n* = 3 (9.6%), *P. luteola* (*n* = 4; 12.9%), *E. coli* (*n* = 7; 22.5%), *C. freundii* (*n* = 2; 6.5%), and *S. marcescens* (*n* = 4; 12.9%).

## 4. Discussion

Following the “One Health” approach, which recognises food as a vector for the spread of antibiotic resistance from the environment to humans, this study sought to identify fresh produce on different farms based on the production and agricultural systems in Free State Province, South Africa, to assess the presence of antibiotic resistance. The nine antimicrobial drugs tested in the present study are widely utilised to treat bacterial infections in livestock and human health.

In this study, *E. coli* (18.4%), *B. cepacia* (15.8%), *P. luteola* (15.8%), and *S. aureus* (13.2%) isolates from cabbage and spinach from both farms and retailers displayed high levels of resistance to most of the antibiotics utilised, while *E. cloacae*, *M. luteus*, *S. sciuri*, *A. haemolyticus*, and *C. freundii* [each 5.3%] showed the least. *Escherichia coli* isolates from the spinach farm displayed high levels of resistance to most of the antibiotics utilised compared *E. coli* isolated from the cabbage farm. In another study on Chinese cabbage, isolates (100%) showed high resistance levels to penicillin but varying resistant characteristics for tetracycline, ampicillin, and amoxicillin, with resistance rates of 31.3% (30/96), 31.3% (30/96), and 31.3% (30/96), respectively [32]. Similarly, other studies have reported higher rates of *E. coli* contamination and resistance in fresh salad vegetables in Pakistan (32.4%) and Nigeria (24.4%), respectively [33,34]. The presence of *E. coli* in food indicates possible contamination from soil, manure, irrigation water, or livestock faeces or may be either directly or indirectly from farm personnel due to poor hygiene. The frequent isolation of *S. aureus* in vegetables has been noted in previous studies [35,36]. In the current study, all *S. aureus* (100%) isolates were susceptible to vancomycin. This is beneficial because vancomycin is the recommended antibiotic for treating MRSA infections, and the appearance of VRSA in vegetables is a concern [36].

The *B. cepacia* isolates (15.8%) from commercially available spinach showed resistance to most tested antibiotics. Similarly, in a study conducted in the USA, all *B. cepacia* isolates were resistant to ceftriaxone, and five isolates were resistant to cefepime, colistin-sulfate, and erythromycin [37]. It is reported that *B. cepacian* raises important ecological issues, including the evolution of pathogenicity and multi-resistant environmental bacteria through horizontal gene transfer, and it is now considered an opportunist human pathogen causing respiratory and urinary tract infections, including bacteremia, in humans [38].

*Haemolytica* spp. are bacterial pathogen most frequently isolated from cattle, and the prevalence of antimicrobial resistance in this pathogen has been increasing [39]. In this study, *A. haemolytica* isolates from retail spinach and cabbage also displayed 100% resistance to tetracycline, ampicillin, ciprofloxacin, and erythromycin, including penicillin. There are no similar cases in the literature to support this hypothesis.

*Serratia marcescens* typically exhibit antibiotic resistance through the production of the enzymes lipase, gelatinase, and deoxyribonuclease (DNase) [40]. All *S. marcescens* isolated from spinach and cabbage farms also showed 100% resistance to chloramphenicol, tetracycline, ciprofloxacin, erythromycin, and penicillin. A recent systematic review found that *S. marcescens* is resistant to a wide range of antibiotics, including penicillin, cephalosporin, tetracycline, macrolide, nitrofurantoin, and colistin, and pointed out that carbapenem should be included in the treatment of *S. marcescens* infections [41]. According to the literature, *S. marcescens* is resistant to a variety of antibiotics, including tetracycline, penicillin, macrolide, nitrofurantoin, colistin, and cephalosporin [40].

In this study, *P. luteola* isolates from cabbage and spinach from both farm and retail isolates displayed high levels of resistance to most of the antibiotics utilised. According to some previous studies, *P. luteola* exhibits high resistance to trimethoprim–sulfamethoxazole, ceftriaxone, tetracycline, and ampicillin [42]. *Pseudomona luteola* has been shown to be resistant to trimethoprim–sulfamethoxazole, ampicillin, tetracycline, and first- and second-generation cephalosporins [43].

The *S. sciuri* isolates from spinach and cabbage farms showed the least 5.3% antibiotic resistance in this study. As a food-borne bacteria, *S. sciuri* spreads easily in street food markets [44,45] and causes spoilage of dairy products, fruits, and vegetables [45]. To date, over 100 *Staphylococcus sciuri* isolates have been characterised, and it has been found that they all carried a genetic element (*S. sciuri mecA*) that is closely related to the *mecA* gene of methicillin-resistant *Staphylococcus aureus* (MRSA) strains [46].

The isolates obtained from this study showed resistance to several antibiotics tested, with 79% of the isolates showing multidrug resistance (MDR). This result is higher than the results of previous studies in Nepal, South Africa, and Switzerland, in which 56.9% (from chutney), 40.3% (from fresh vegetables), and 20.5% (from fresh produce) of isolates were MDR [13,47,48]. In contrast, a study conducted in Bangladesh reported a high proportion (98.06%) of isolates with MDR from raw salad vegetables [49]. The presence of MDR in isolates from fresh vegetables must be taken seriously, as they act as a reservoir and can potentially transmit the bacteria to humans.

The utilisation of antibiotics in animal husbandry and the simultaneous spread of antibiotic-resistant bacteria in manure mean that these bacteria can persist in agricultural soils [37,50]. Soil can be considered a large reservoir of antibiotic resistance determinants since it is present in all plants, small animals, fungi, protists, and soil bacteria [51,52]. In addition, the cross-contamination of fruits and vegetables after harvest and horizontal gene transfer may contribute to this situation [36]. The recently published review has shown that it is difficult to disinfect contaminated vegetables, especially when the bacteria have been absorbed from the soil and established themselves in the plant tissue [52]. This study has several notable limitations, including a small sample size, a limited variety of vegetables, and the absence of screening for antibiotic-resistant genes.

## 5. Conclusions

This is the first study to demonstrate antimicrobial resistance in bacteria isolated from fresh vegetables in Free State Province, South Africa. The literature depicts leafy green vegetables as a reservoir for multidrug-resistant pathogens and commonly implicated in disease outbreaks worldwide. Plant uptake and the bioaccumulation of antibiotics draw attention to the need for better food safety practices in the supply chain and the identification of sources of contamination of fresh produce with antibiotic-resistant bacteria as a public health concern. The isolates from this study demonstrated high resistance characteristics to multiple antibiotic classes, including *β*-lactams, chloramphenicol, tetracycline, aminoglycosides, and macrolides, mostly from farm origin. To ensure safe fresh vegetable production and distribution, minimising antibiotic-resistant bacteria risk is crucial. Additionally, regulated parties must oversee and promote safe handling practices throughout the production chain. The AR profile comparisons across vegetables can guide future mitigation strategies.

## Figures and Tables

**Table 1:** Identification of different bacteria isolated from spinach and cabbage.

Retail																				
**Spinach**											**Cabbage**									
	EC1	SA	ML	SS	AH	BC	PL	EC2	CF	SM	EC1	SA	ML	SS	AH	BC	PL	EC2	CF	SM
**R1**	-	-	-	-	1	-	1	-	-	-	-	-	-	-	1	-	1	-	-	-
**R2**	1	-	-	-	-	-	-	1	-	-	-	-	-	-	-	-	-	-	-	-
**R3**	-	-	-	-	-	-	-	-	-	-	1	-	-	-	-	1	-	1	-	-
**R4**	-	-	-	-	-	1	1	-	-	-	-	-	-	-	-	-	-	-	-	-
**R5**	-	-	-	-	-	1	1	1	-	-	-	-	-	-	-	1	-	-	-	-
**Farms**																				
**F1**	-	1	-	-	-	-	-	-	-	-	-	-	-	-	-	-	1	-	-	1
**F2**	-	1	1	-	-	1	1	1	1	1	-	-	1	-	-	-	-	1	-	-
**F3**	-	-	-	-	-	-	-	-	-	-	-	-	-	1	-	-	-	-	-	-
**F4**	-	1	-	1	-	-	-	1	1	-	-	1	-	-	-	1	-	-	-	1
**F5**	-	-	-	-	-	-	-	-	-	1	-	1	-	-	-	-	-	1	-	-

*E. cloacae* = EC1, *S. aureus* (SA), *M. luteus* (ML), *S. sciuri* (SS), *A. haemolyticus* (AH), *B. cepacia* (BC), *P. luteola* (PL), *E. coli* (EC2), *C. freundii* (CF), and *S. marcescens* (SM).

**Table 2:** The antibiotic resistance profiles of the isolates from samples of spinach and cabbage.

Retails																				
**Spinach**											**Cabbage**									
Ant	EC1	SA	ML	SS	AH	BC	PL	EC2	CF	SM	EC1	SA	ML	SS	AH	BC	PL	EC2	CF	SM
C	1	-	-	-	-	1	1	2	-	-	-	-	-	-	-	-	1	1	-	-
TE	1	-	-	-	1	1	2	2	-	-	1	-	-	-	1	1	1	1	-	-
CN	-	-	-	-	-	1	-	2	-	-	-	-	-	-	-	1	-	1	-	-
AMP	1	-	-	-	1	1	2	1	-	-	1	-	-	-	1	-	1	1	-	-
CIP	-	-	-	-	1	2	-	-	-	-	-	-	-	-	1	1	-	-	-	-
E	-	-	-	-	1	1	-	2	-	-	-	-	-	-	1	-	-	1	-	-
CAZ	1	-	-	-	-	-	-	2	-	-	-	-	-	-	-	-	-	1	-	-
VA	-	-	-	-	1	-	-	-	-	-	-	-	-	-	1	-	-	-	-	-
P	-	-	-	-	1	1	3	1	-	-	-	-	-	-	1	-	1	1	-	-
**Farms**																				
**Spinach**											**Cabbage**									
C	-	-	1	-	-	1	1	2	-	2	-	-	1	-	-	1	1	2	-	2
TE	-	3	-	-	-	1	1	2	2	2	-	2	-	-	-	1	1	2	-	2
CN	-	3	1	1	-	2	-	2	2	-	-	2	1	-	-	1	-	2	-	-
AMP	-	3	1	-	-	1	3	2	2	-	-	2	1	-	-	-	1	1	-	-
CIP	-	2	-	-	-	1	-	-	2	2	-	1	-	-	-	-	-	-	-	2
E	-	2	-	1	-	1	-	2	-	2	-	2	-	-	-	-	-	2	-	2
CAZ	-	-	1	-	-	-	1	2	1	-	-	-	1	-	-	-	-	1	-	-
VA	-	-	-	-	-	-	-	-	-	-	-	-	-	-	-	-	-	-	-	-
P	-	3	1	1	-	-	1	2	-	2	-	2	1	1	-	-	1	1	-	2

*E. cloacae* = EC1, *S. aureus* (SA), *M. luteus* (ML), *S. sciuri* (SS), *A. haemolyticus* (AH), *B. cepacia* (BC), *P. luteola* (PL), *E. coli* (EC2), *C. freundii* (CF), *S. marcescens* (SM), Antibiotic (Ant).

**Table 3:** Resistance patterns of bacterial isolates from farm and retail leafy vegetable samples.

Species	No. of Isolates	C	TE	CN	AMP	CIP	E	CAZ	VA	P
*E. cloacae*	2	1 (50%)	2 (100%)	-	2 (100%)	-	-	1 (50%)	-	-
*S. aureus*	5	-	5 (100%)	5 (100%)	5 (100%)	3 (60%)	4 (80%)	-	-	5 (100%)
*M. luteus*	2	2 (100%)	-	2 (100%)	2 (100%)	-	-	2 (100%)	-	2 (100%)
*S. sciuri*	2	-	-	1 (50%)	-	-	1 (50%)	-	-	2 (100%)
*A. haemolyticus*	2	-	2 (100%)	-	2 (100%)	2 (100%)	2 (100%)	-	2 (100%)	2 (100%)
*B. cepacia*	6	2 (33%)	4 (67%)	5 (83%)	2 (33%)	3 (50%)	2 (33%)	-	-	1 (17%)
*P. luteola*	6	4 (67%)	6 (100%)	-	5 (83%)	-	-	1 (17%)	-	6 (100%)
*E. coli*	7	7 (100%)	7 (100%)	7 (100%)	6 (86%)	-	7 (100%)	6 (86%)	-	5 (71%)
*C. freundii*	2	-	2 (100%)	2 (100%)	2 (100%)	2 (100%)	-	1 (50%)	-	-
*S. marcescens*	4	4 (100%)	4 (100%)	-	-	4 (100%)	4 (100%)	-	-	4 (100%)

C = chloramphenicol, TE = tetracycline, CN = gentamicin, AMP = ampicillin, CIP = ciprofloxacin, E = erythromycin, CAZ = ceftazidime, VA = vancomycin, P = penicillin.

## Data Availability

The original contributions presented in this study are included in the article. Further inquiries can be directed to the corresponding author.

## References

[1] Djordjevic S.P., Jarocki V.M., Seemann T., Cummins M.L., Watt A.E., Drigo B., Wyrsch E.R., Reid C.J., Donner E., Howden B.P. (2024). Genomic surveillance for antimicrobial resistance—A One Health perspective. Nat. Rev. Genet..

[2] Ahmad N., Joji R.M., Shahid M. (2023). Evolution, and implementation of one health to control the dissemination of antibiotic-resistant bacteria and resistance genes: A review. Front. Cell. Infect Microbiol..

[3] Verraes C., Van Boxstael S., Van Meervenne E., Van Coillie E., Butaye P., Catry B., de Schaetzen M.A., Van Huffel X., Imberechts H., Dierick K. (2013). Antimicrobial resistance in the food chain: A review. Int. J. Environ. Res. Public Health.

[4] Mohan A., Bashir S., Mohan A., Kumar D., Kaur N. (2023). Occurrence and Fate of Antibiotics in Manure. Manure Technology and Sustainable Development.

[5] Swinkels A.F., Berendsen B.J., Fischer E.A., Zomer A.L., Wagenaar J.A. (2024). Extended period of selection for antimicrobial resistance due to recirculation of persistent antimicrobials in broilers. J. Antimicrob. Chemother..

[6] Lopez-Velasco G., Welbaum G.E., Boyer R.R., Mane S.P., Ponder M.A. (2011). Changes in spinach phylloepiphytic bacteria communities following minimal processing and refrigerated storage described using pyrosequencing of 16S rRNA amplicons. J. Appl. Microbiol..

[7] Joy S.R., Bartelt-Hunt S.L., Snow D.D., Gilley J.E., Woodbury B.L., Parker D.B., Marx D.B., Li X. (2013). Fate and transport of antimicrobials and antimicrobial resistance genes in soil and runoff following land application of swine manure slurry. Environ. Sci. Technol..

[8] Sandberg K.D., LaPara T.M. (2016). The fate of antibiotic resistance genes and class 1 integrons following the application of swine and dairy manure to soils. FEMS Microbiol. Ecol..

[9] Cornejo-Juárez P., Vilar-Compte D., Pérez-Jiménez C., Namendys-Silva S.A., Sandoval-Hernández S., Volkow-Fernández P. (2015). The impact of hospital-acquired infections with multidrug-resistant bacteria in an oncology intensive care unit. Int. J. Infect. Dis..

[10] Pulingam T., Parumasivam T., Gazzali A.M., Sulaiman A.M., Chee J.Y., Lakshmanan M., Chin C.F., Sudesh K. (2022). Antimicrobial resistance: Prevalence, economic burden, mechanisms of resistance and strategies to overcome. Eur. J. Pharm. Sci..

[11] Salam M.T., Bari K.B., Rahman M.M., Gafur D.M.M., Faruk M.O., Akter K., Irin F., Ashakin M.R., Shaikat T.A., Das A.C. (2024). Emergence of antibiotic-resistant infections in ICU patients. J. Angiother..

[12] Rahman M., Alam M.U., Luies S.K., Kamal A., Ferdous S., Lin A., Sharior F., Khan R., Rahman Z., Parvez S.M. (2021). Contamination of fresh produce with antibiotic-resistant bacteria and associated risks to human health: A scoping review. Int. J. Environ. Res. Public Health.

[13] Kläui A., Bütikofer U., Naskova J., Wagner E., Marti E. (2024). Fresh produce as a reservoir of antimicrobial resistance genes: A case study of Switzerland. Sci. Total Environ..

[14] Verhaegen M., Bergot T., Liebana E., Stancanelli G., Streissl F., Mingeot-Leclercq M.P., Mahillon J., Bragard C. (2023). On the use of antibiotics to control plant pathogenic bacteria: A genetic and genomic perspective. Front. Microbiol..

[15] Endale H., Mathewos M., Abdeta D. (2023). Potential causes of spread of antimicrobial resistance and preventive measures in One Health perspective—A review. Infect. Drug Resist..

[16] Azanu D., Mortey C., Darko G., Weisser J.J., Styrishave B., Abaidoo R.C. (2016). Uptake of antibiotics from irrigation water by plants. Chemosphere.

[17] Gudda F., Odinga E.S., Tang L., Waigi M.G., Wang J., Abdalmegeed D., Gao Y. (2023). Tetracyclines uptake from irrigation water by vegetables: Accumulation and antimicrobial resistance risks. Environ. Pollut..

[18] Van T.T.H., Yidana Z., Smooker P.M., Coloe P.J. (2020). Antibiotic use in food animals worldwide, with a focus on Africa: Pluses and minuses. J. Glob. Antimicrob. Resist..

[19] Centers for Disease Control and Prevention (2019). National Center for Emerging and Zoonotic Infectious Diseases (NCEZID), Division of Healthcare Quality Promotion (DHQP). https://www.cdc.gov/antimicrobial-resistance/data-research/threats/index.html.

[20] Colomb-Cotinat M., Lacoste J., Brun-Buisson C., Jarlier V., Coignard B., Vaux S. (2016). Estimating the morbidity and mortality associated with infections due to multidrug-resistant bacteria (MDRB), France. Antimicrob. Resist. Infect. Control.

[21] Elton L., Thomason M.J., Tembo J., Velavan T.P., Pallerla S.R., Arruda L.B., Vairo F., Montaldo C., Ntoumi F., Hamid M.M.A. (2020). Antimicrobial resistance preparedness in sub-Saharan African countries. Antimicrob. Resist. Infect. Control.

[22] Phares C.A., Danquah A., Atiah K., Agyei F.K., Michael O.T. (2020). Antibiotics utilization and farmers’ knowledge of its effects on soil ecosystem in the coastal drylands of Ghana. PLoS ONE.

[23] Tadesse B.T., Ashley E.A., Ongarello S., Havumaki J., Wijegoonewardena M., González I.J., Dittrich S. (2017). Antimicrobial resistance in Africa: A systematic review. BMC Infect. Dis..

[24] Msimango T., Duvenage S., Du Plessis E.M., Korsten L. (2023). Microbiological quality assessment of fresh produce: Potential health risk to children and urgent need for improved food safety in school feeding schemes. Food Sci. Nutr..

[25] Njage P.M., Buys E.M. (2015). Pathogenic and commensal *Escherichia coli* from irrigation water show potential in transmission of extended spectrum and AmpC β-lactamases determinants to isolates from lettuce. Microb. Biotechnol..

[26] Ratshilingano M.T., du Plessis E.M., Duvenage S., Korsten L. (2022). Characterization of multidrug-resistant *Escherichia coli* isolated from two commercial lettuce and spinach supply chains. J. Food Prot..

[27] Richter L., Du Plessis E.M., Duvenage S., Korsten L. (2019). Occurrence, identification, and antimicrobial resistance profiles of extended-spectrum and AmpC β-lactamase-producing Enterobacteriaceae from fresh vegetables retailed in Gauteng Province, South Africa. Foodborne Pathog. Dis..

[28] Mohapi D., Nkhebenyane S., Khetsha Z., Thekisoe O. (2024). Phyllo-epiphytic and endophytic pathogens on *Brassica oleracea* Var. *capitata* L. and *Spinacia oleracea* L. as affected by small-scale farm production systems. Appl. Ecol. Environ. Res..

[29] Ramatla T., Tutubala M., Motlhaping T., de Wet L., Mokgokong P., Thekisoe O., Lekota K. (2024). Molecular detection of Shiga toxin and extended-spectrum beta-lactamase (ESBL)-producing *Escherichia coli* isolates from sheep and goats. Mol. Biol. Rep..

[30] CLSI (Clinical and Laboratory Standards Institute) (2020). Performance Standards for Antimicrobial Susceptibility Testing.

[31] CLSI (Clinical and Laboratory Standards Institute) (2023). Performance Standards for Antimicrobial Disk and Dilution Susceptibility Tests for Bacteria Isolated from Animals.

[32] Datta S., Ishikawa M., Chudhakorn S., Charaslertrangsi T. (2024). Prevalence and antimicrobial characteristics of *Escherichia coli* in selected vegetables and herbs in Bangkok, Thailand. J. Food Prot..

[33] Shah M.S., Eppinger M., Ahmed S., Shah A.A., Hameed A., Hasan F. (2015). Multidrug-resistant diarrheagenic *E. coli* pathotypes are associated with ready-to-eat salad and vegetables in Pakistan. J. Korean Soc. Appl. Biol. Chem..

[34] Igbinosa E.O., Beshiru A., Igbinosa I.H., Cho G.S., Franz C.M. (2023). Multidrug-resistant extended spectrum β-lactamase (ESBL)-producing Escherichia coli from farm produce and agricultural environments in Edo State, Nigeria. PLoS ONE.

[35] Seo Y.H., Jang J.H., Moon K.D. (2010). Occurrence and characterization of enterotoxigenic *Staphylococcus aureus* isolated from minimally processed vegetables and sprouts in Korea. Food Sci. Biotechnology.

[36] Jia K., Qin X., Bu X., Zhu H., Liu Y., Wang X., Li Z., Dong Q. (2023). Prevalence, antibiotic resistance and molecular characterization of *Staphylococcus aureus* in ready-to-eat fruits and vegetables in Shanghai, China. Curr. Res. Food Sci..

[37] Karumathil D.P., Yin H.B., Kollanoor-Johny A., Venkitanarayanan K. (2016). Prevalence of multidrug-resistant bacteria on fresh vegetables collected from farmers’ markets in Connecticut. J. Food Prot..

[38] Sousa S.A., Ramos C.G., Leitao J.H. (2011). *Burkholderia cepacia* complex: Emerging multi-host pathogens equipped with a wide range of virulence factors and determinants. Int. J. Microbiol..

[39] Snyder E., Credille B., Berghaus R., Giguère S. (2017). Prevalence of multi drug antimicrobial resistance in *Mannheimia haemolytica* isolated from high-risk stocker cattle at arrival and two weeks after processing. J. Anim. Sci..

[40] Zivkovic Zaric R., Zaric M., Sekulic M., Zornic N., Nesic J., Rosic V., Vulovic T., Spasic M., Vuleta M., Jovanovic J. (2023). Antimicrobial treatment of Serratia marcescens invasive infections: Systematic review. Antibiotics.

[41] Cosimato I., Santella B., Rufolo S., Sabatini P., Galdiero M., Capunzo M., Boccia G., Folliero V., Franci G. (2024). Current Epidemiological Status and Antibiotic Resistance Profile of Serratia marcescens. Antibiotics.

[42] Ahmad S., Alzahrani A.J., Alsaeed M. (2023). Uncommon association: *Pseudomonas luteola* bacteremia in an immunocompetent individual with acute tonsillitis—A case report. IDCases.

[43] Yousefi F., Shoja S., Honarvar N. (2014). Empyema caused by *Pseudomonas luteola*: A case report. Jundishapur J. Microbiol..

[44] Yang T.Y., Hung W.W., Lin L., Hung W.C., Tseng S.P. (2017). *mecA*-related structure in methicillin-resistant coagulase-negative staphylococci from street food in Taiwan. Sci. Rep..

[45] Makky S., Abdelsattar A.S., Habashy M., Dawoud A., Nofal R., Hassan A., Connerton I.F., El-Shibiny A. (2023). Phage ZCSS1 from isolation to application against *Staphylococcus sciuri* and biofilm: A prospect of utilizing temperate phage and its products. Gene Rep..

[46] Couto I., Sanches I.S., Sá-Leão R., de Lencastre H. (2000). Molecular characterization of *Staphylococcus sciuri* strains isolated from humans. J. Clin. Microbiol..

[47] Adhikari S., Regmi R.S., Sapkota S., Khadka S., Patel N., Gurung S., Thapa D., Bhattarai P., Sapkota P., Devkota R. (2023). Multidrug resistance, biofilm formation and detection of *bla*_CTX-M_ and *bla*_VIM_ genes in *E. coli* and *Salmonella* isolates from chutney served at the street-food stalls of Bharatpur, Nepal. Heliyon.

[48] Richter L., Plessis E.D., Duvenage S., Korsten L. (2021). High prevalence of multidrug resistant *Escherichia coli* isolated from fresh vegetables sold by selected formal and informal traders in the most densely populated Province of South Africa. J. Food Sci..

[49] Nipa M.N., Mazumdar R.M., Hasan M.M., Fakruddin M.D., Islam S., Bhuiyan H.R., Iqbal A. (2011). Prevalence of multi drug resistant bacteria on raw salad vegetables sold in major markets of Chittagong city, Bangladesh. Middle-East J. Sci. Res..

[50] Sarmah A.K., Meyer M.T., Boxall A.B. (2006). A global perspective on the use, sales, exposure pathways, occurrence, fate and effects of veterinary antibiotics (VAs) in the environment. Chemosphere.

[51] Monier J.M., Demanèche S., Delmont T.O., Mathieu A., Vogel T.M., Simonet P. (2011). Metagenomic exploration of antibiotic resistance in soil. Curr. Opin. Microbiol..

[52] Nkhebenyane S.J., Khasapane N.G., Lekota K.E., Thekisoe O., Ramatla T. (2024). Insight into the prevalence of extended-spectrum *β*-lactamase-producing Enterobacteriaceae in vegetables: A Systematic review and meta-analysis. Foods.

